# Membrane-Active Antibiotics Affect Domains in Bacterial Membranes as the First Step of Their Activity

**DOI:** 10.1021/acs.nanolett.4c01873

**Published:** 2024-08-15

**Authors:** Adéla Melcrová, Christiaan Klein, Wouter H. Roos

**Affiliations:** † Molecular Biophysics, Zernike Institute for Advanced Materials, Rijksuniversiteit Groningen, 9712 AG Groningen, The Netherlands

**Keywords:** antimicrobial resistance, bacterial membrane, atomic force microscopy, antimicrobial agent

## Abstract

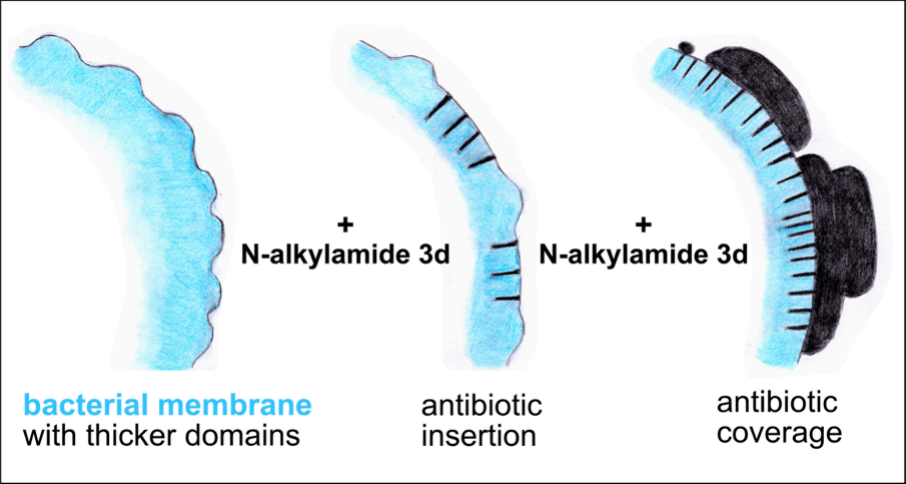

The need to combat antimicrobial resistance is becoming more and more pressing. Here we investigate the working mechanism of a small cationic agent, *N*-alkylamide 3d, by conventional and high-speed atomic force microscopy. We show that *N*-alkylamide 3d interacts with the membrane of *Staphylococcus aureus*, where it changes the organization and dynamics of lipid domains. After this initial step, supramolecular structures of the antimicrobial agent attach on top of the affected membrane gradually, covering it entirely. These results demonstrate that lateral domains in the bacterial membranes might be affected by small antimicrobial agents more often than anticipated. At the same time, we show a new dual-step activity of *N*-alkylamide 3d that not only destroys the lateral membrane organization but also effectively covers the whole membrane with aggregates. This final step could render the membrane inaccessible from the outside and possibly prevent signaling and waste disposal of living bacteria.

Antimicrobial resistance has been declared by the World Health Organization as one of the top 10 global threats for medical care.^1^ Worldwide, almost 5 million deaths per year are associated with antimicrobial-resistant bacteria.^2^ Membrane-active peptides and peptide-like mimics without a specific protein target could help us to overcome the antimicrobial resistance crisis, especially concerning Gram-positive bacteria such as *Staphylococcus aureus*, whose envelope consists of only a single membrane and a peptidoglycan cell wall. These antibiotics target the membranes that are specific for bacteria, i.e., consisting of a high amount of negatively charged lipids and of lipids with branched acyl tails.^3,4^ Bacteria do have a defense mechanism in which they can become less susceptible to certain membrane-active peptides by decreasing the overall negative charge or by replacing some of the iso-branched lipids with anteiso-branched. However, these bacterial membranes still remain clearly distinct from human cell membranes, as overall bacterial membranes are highly conserved.^4,5^ Recently, we reported about a peptide-like mimic antibiotic, AMC-109,^6^ which incorporates in between bacterial lipids, changing their lateral organization and dissolving the membrane domains in *S. aureus* lipid extracts. The lateral organization of the bacterial membrane is a promising antimicrobial target, as the bacteria cannot function without the functional membrane microdomains,^7^ which have an irreplaceable role in protein sorting, bacteria cell signaling, and building of the cell wall. Moreover, it was reported that methicillin-resistant *S. aureus* that is mutated to grow without the functional membrane microdomains loses its resistance to β-lactam antibiotics.^8^ In addition to AMC-109^6^ also daptomycin^9,10^ was shown to target membrane domains. Overall, the selectivity of positively charged antibiotics toward bacteria is driven by the negative charge of the bacterial surface and leads to generally low cytotoxicity and resistance generation.

Atomic force microscopy (AFM) is a single-particle technique that allows for studying molecular level interactions of antibiotics with bacteria and supported membranes.^11,12^ Conventional AFM has been previously shown to distinguish various effects of antimicrobial molecules on membranes, including membrane thinning,^13^ disruption,^14,15^ and pore formation.^16^ High-speed AFM adds the possibility of observing the kinetics of antimicrobial activity. This includes dynamic observations of membrane thinning,^17^ pore formation,^6,18^ lipid domain dissolution,^6^ calcium-dependent carpet formation,^19^ and blocking cell wall synthesis.^17,20^ This shows that with the development of high-speed AFM now peptidomimetic-induced changes of bacterial membranes can be observed with subsecond resolution.

Here we report on the recently described peptidomimetic *N*-alkylamide 3d, a dimeric molecule combining the properties of a lipid and a peptide^21^ (Figure 1a). *N*-Alkylamide 3d is an antibiotic molecule that was specifically designed to target bacterial lipid membranes. It has a broad range of activity against both Gram-positive and Gram-negative bacteria strains with minimal inhibitory concentrations (MIC) ranging between 0.75 and 6 μg/mL for various pathogens, while displaying cytotoxicity only at 2 orders of magnitude higher concentrations.^21^ Using conventional and high-speed atomic force microscopy,^6,17,18,22^ we study the mode of action of *N*-alkylamide 3d and reveal in time how it targets bacterial membrane domains. The activity of *N*-alkylamide 3d starts with affecting the membrane domain mobility and domain dissolution. This is followed by the appearance of small aggregates of this antibiotic and finally formation of carpets and rod-like supramolecular structures on the membrane surface. Our results indicate that membrane-active antibiotics could affect the bacterial membrane domains more often than anticipated.

**Figure 1:**
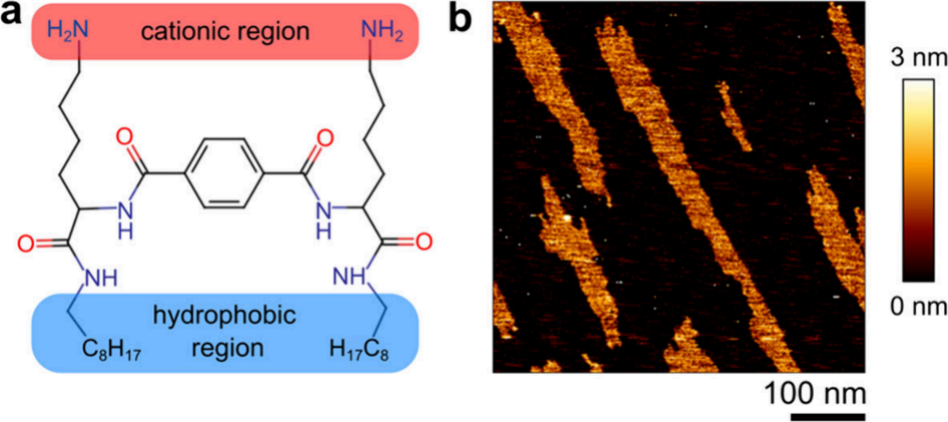
*N*-Alkylamide 3d directional growth on mica. (a) Chemical structure of *N*-alkylamide 3d. Adapted with permission from ref (21). Copyright 2018 American Chemical Society. (b) An example image of *N*-alkylamide 3d attaching onto a mica surface obtained by conventional AFM, 20 min after the addition of 2 μg/mL *N*-alkylamide 3d.

To observe the activity of *N*-alkylamide 3d on the membranes, we employed AFM imaging on bacterial membranes supported on mica. To test whether there are also interactions with the support, instead of only with the membranes, we imaged the clean mica in a PBS buffer, to which we then added *N*-alkylamide 3d in concentrations of 1–10 μg/mL. We observe attachment of *N*-alkylamide 3d molecules to mica and then epitaxial growth that always follows a single direction (Figures 1b and S1). Epitaxial growth of peptides and other biomolecules along the hexagonal lattice of mica^23^ is used to engineer highly ordered self-assembled biomimetic materials and fabricate nanopatterns on surfaces for molecular devices,^24−26^ to study self-assembly of amyloid-like structures,^27,28^ or to study specific interactions between biomolecules and inorganic surfaces.^29,30^ In our case, *N*-alkylamide 3d displays an unusual single-directional growth that was observed for biomolecules at only a few instances,^25,26^ instead of three-directional growth, as is more commonly observed.^24,27−30^

In an attempt to avoid these interactions with the support, we tested the *N*-alkylamide 3d interaction with other surfaces as well as the stability of the full lipid extracts from *Staphylococcus aureus* bacteria—*S. aureus* lipid membranes—on these surfaces. In particular, gold Au(111), poly-l-lysine-coated glass, SiO_2_-coated silicon wafers, and plasma-cleaned glass were tested. We did not observe epitaxial growth of *N*-alkylamide 3d on gold Au(111) (Figure S2). However, this surface stabilizes the membranes in the form of vesicles (Figure S3b) and does not support the formation of supported lipid bilayers. Therefore, for the other surfaces we first tested the compatibility of the surface with the membrane. Poly-l-lysine-coated glass also stabilized the vesicles (Figure S3a) and did not lead to the formation of a supported lipid bilayer, in accordance with previous results.^31^ The supported membranes on SiO_2_-coated silicon wafers appear to be fractured and not stable (Figure S3d), making this surface also unsuitable. The hydrophilic nature of the plasma-cleaned glass leads to fast spreading and possibly thinning of the supported membranes. In this case, we cannot verify the thickness and stability of the membrane, as we do not observe the membrane edge (Figure S3e). So finally only the mica surface leads to proper membranes (Figure S3c). In this case, the membranes are stable with a thickness of ∼6 nm on the top of the lateral domains corresponding to a single supported membrane (Figure S4). We, hence, optimized the preparation of the membranes on this surface, in a manner where the majority of the mica surface is covered with the membrane. This way we minimize interactions of the *N*-alkylamide 3d with the bare mica. In Figure S4 it can be seen that the membrane domains are blurry. Therefor a faster imaging technique than conventional AFM is needed, and we turn to high-speed AFM.^32−34^ This allows for (sub)second imaging (here we usually image with a rate of 1–2 s per frame), which means we image faster than the domain movement. Furthermore, it allows us to observe antibiotic growth on the surrounding support, which can be accounted for during data analysis. High-speed AFM proved instrumental in the past to be aware of height differences in the background that affect the proper interpretation of the antibiotic activity on membranes. Using high-speed AFM we previously revealed attachment of micellar antibiotic aggregates around the studied membranes,^6^ movement of membrane domains in complex lipid membranes extracted from bacteria,^6^ and growth of antibiotic filaments on supported membranes.^17,20^

## *N*-Alkylamide 3d Affects the Membrane Domains in *S. aureus* Lipid Membrane

**Figure 2:**
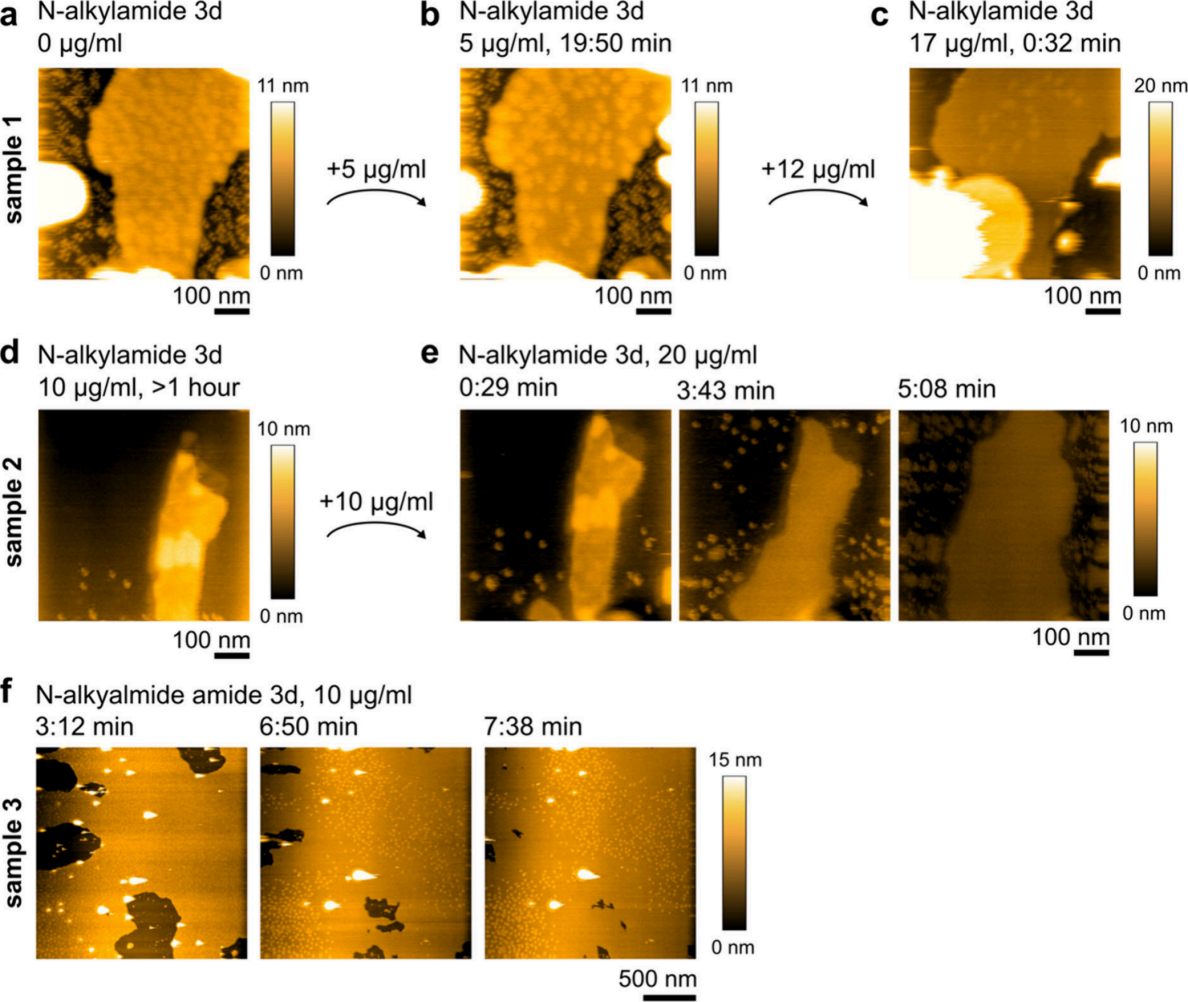
High-speed AFM imaging reveals *N*-alkylamide 3d’s interactions with the *S. aureus* lipid membrane and how the antibiotics affect the membrane domains (bright spots on the membrane). (a) Membrane domains (bright yellow) in an untreated *S. aureus* lipid membrane (dark yellow) are equally distributed and move rapidly. Surrounding mica is depicted in black. White spots are unruptured liposomes. (b) Incorporation of *N*-alkylamide 3d into the membrane slows down the movement of membrane domains and the domains start clustering. (a, b) Snapshots from Supporting Video 1. See the video to follow the domain movement in time. (c) Further *N*-alkylamide 3d addition leads to dissolution of the domains, spread of the membrane, and eventually attachment of larger aggregates (white in lower left corner) and carpets (bright yellow) on top of the membrane. This step-by-step activity of *N*-alkylamide 3d was observed in 6 independent high-speed AFM experiments. (d, e) Another example of the *S. aureus* lipid membrane with clustered domains after exposure to 10 μg/mL *N*-alkylamide 3d. Increase in the *N*-alkylamide 3d concentration to 20 μg/mL (e) leads to dissolution of the domains and spread of the membrane. The additional patches at panel e at 3:43 min likely correspond to the attachment of *N*-alkylamide 3d directly to mica as in Figure 1b and Figure S1. These initially small patches then grow laterally in size (e = 5:08 min). (f) Third example of the *S. aureus* lipid membrane after the exposure to 10 μg/mL *N*-alkylamide 3d. In this case, the membrane covers the majority of the available surface. 3:12 min after the *N*-alkylamide 3d addition, individual domains are still distinguishable. At 6:50 after the addition, the domains stopped their movement (they are in the same position as at 7:38 min) and the membrane started spreading. The spreading continues until full mica coverage is reached. The time stamps in [min:s] denote the time after the addition of the stated antibiotic concentration.

Using full lipid extracts from *S. aureus*, the activity of *N*-alkylamide 3d on these complex lipid membranes was followed. As previously reported for the peptidomimetic AMC-109,^6^ high-speed AFM (HS-AFM) was used in order to follow dynamic changes. The *S. aureus* lipid membranes contain mainly glycerophosholipids, i.e., phosphatidylglycerol (PG), its lysinated version, lysyl-PG, and cardiolipin (CL), and glycolipids, i.e., diglycosyldiacylglycerol and monoglycosyldiacylglycerol.^6^ This membrane composition leads to the formation of stable lateral domains that move rapidly within the membrane (Supporting Video 1). Figure 2a shows the *S. aureus* lipid membrane (yellow) supported on mica (black). Membrane domains are visible as bright yellow spots equidistantly distributed throughout the membrane area. Addition of *N*-alkylamide 3d leads to a slowdown of domain movement (Figure 2b, d, and f, Supporting video 1), their disappearance, and gradual spread of the membrane (Figure 2c and e). The movement of individual domains in the untreated *S. aureus* lipid membranes is faster than our time resolution of 1 s per frame. After the *N*-alkylamide addition, we can distinguish the slowed movement of the individual domains (Supporting Video 1) or even observe their complete halt (Figure 2f). The disappearance of the domains was observed over the whole tested range of 5–20 μg/mL of *N*-alkylamide 3d. We analyzed the rate of the domains’ disappearance on an example of sample 1 in Figure 2a–c (Figure S5). Before the antibiotic treatment, the domains occupy ∼52% of the membrane area; 20 min after the addition of 5 μg/mL *N*-alkylamide 3d they appear sharper due to their slower movement, but still occupy ∼48% of the membrane. Increase of the *N*-alkylamide concentration to 17 μg/mL leads to immediate changes. Fifteen seconds after, the domains take up only ∼39% of the membrane area, which drops to ∼24% after an additional 7 s. Imaging quality was often lowered after the addition of *N*-alkylamide 3d due to its attachment to both the surface and the AFM tip. In cases where the imaging quality was lower, the domains were not distinguishable, but still the membrane spread was observed. Such observations suggest a mechanism, in which *N*-alkylamide 3d enters the *S. aureus* lipid membrane and incorporates between the membrane lipids. Similarly to AMC-109, it then changes the lateral organization of the membrane, slowing down and dissolving the membrane domains.^6^*N*-Alkylamide could affect the membrane by peripheral attachment, or it can really be inserted into the membrane. The observed changes in the domains’ dynamic movement favor an interpretation in which they are internalized into the membrane, which will force the membrane lipids to reorganize. Moreover, taking into account the chemistry of the molecule, the insertion of the *N*-alkylamide 3d into a single leaflet in between the lipids is likely, with the two acyl tails sitting among the lipid hydrophobic tails and the amide cationic region being exposed to the bulk water together with surrounding lipid headgroups.

## The Membrane Gets Covered by Supramolecular Aggregates

**Figure 3:**
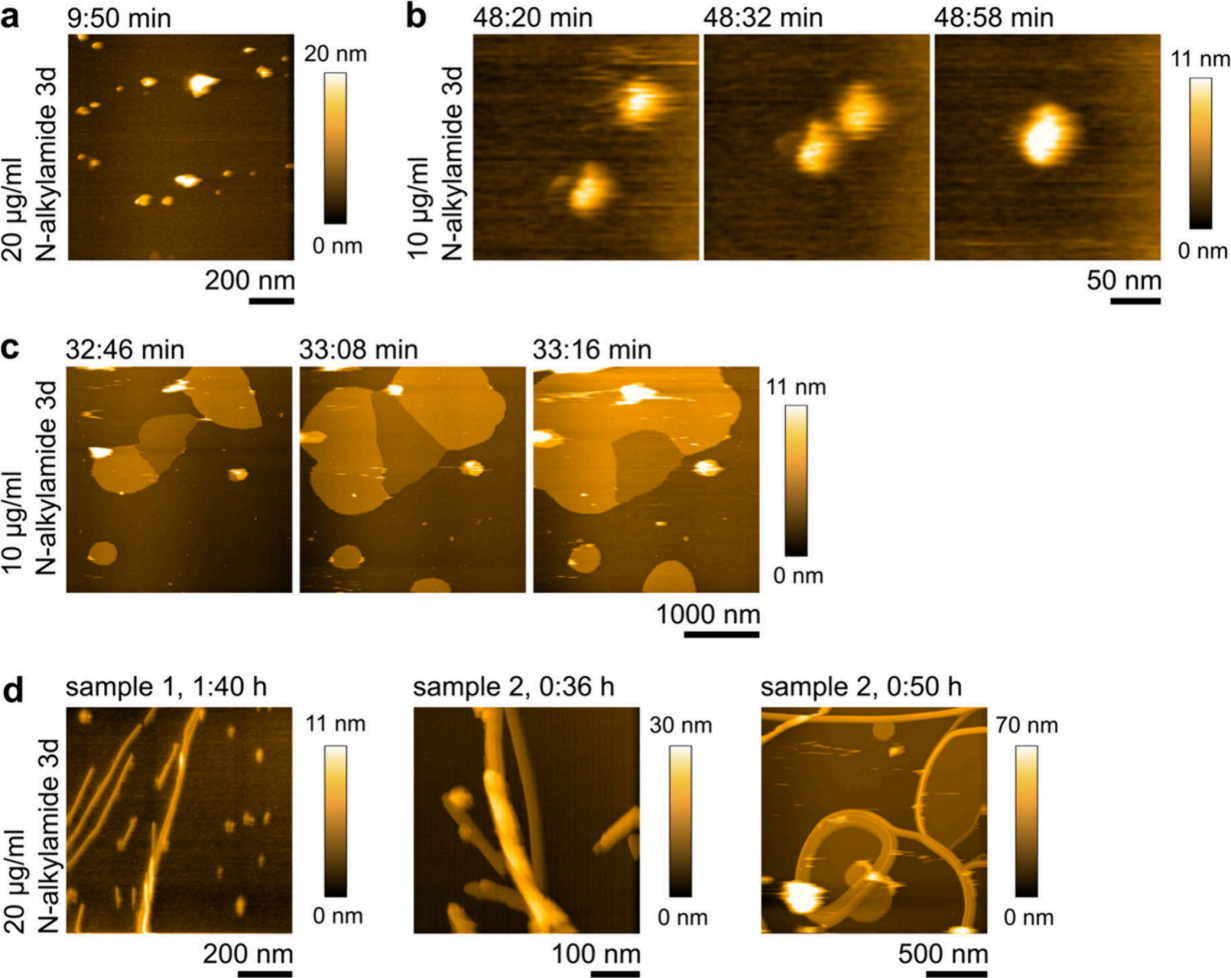
Supramolecular aggregates attach on top of an already spread *S. aureus* lipid membrane recorded by high-speed AFM. The background here is formed by an *S. aureus* lipid membrane that after *N*-alkylamide 3d treatment lost the domains and spread over the whole available mica surface. (a, b) Sphere-shaped aggregates. Similar results were observed in 9 independent high-speed AFM experiments on membranes supported on mica and 4 conventional AFM experiments on membranes supported either on mica or on plasma-cleaned glass. (b) Sphere-shaped aggregates are sometimes mobile on the membrane, and we observe their merging together. (c) Carpets of *N*-alkylamide 3d start forming typically >30 min after the antibiotic addition. Here we observe the growth of a single-layer and a double-layer carpet, each layer ∼6 nm thick. Similar results were observed in 3 independent high-speed experiments on membranes supported on mica and 3 conventional AFM experiments on membranes supported on plasma-cleaned glass. (d) Higher-order structures appear after waiting 30–90 min. We observe formation of rod-like structures that can grow also over each other (left), grow twisted (middle), and be mixed together with the carpets that were reported in panel (c). Three representative examples from 2 independent experiments are shown. Similar results were observed in 6 independent high-speed AFM experiments on membranes supported on mica and 3 AFM experiments on membranes supported on plasma-cleaned glass. Time stamps (a–c) in [min:s] and in (d) [hour:min] denote the time after addition of the stated concentrations of the antibiotic.

Interestingly, the *N*-alkylamide 3d activity does not stop at incorporation into the membrane. After the domain dissolution, the membrane always spreads until it fully covers the whole available mica surface. This behavior again points toward an interpretation in which *N*-alkylamide 3d molecules are being inserted into the *S. aureus* lipid membrane and not affecting it just by peripheral attachment. Their insertion provides additional material to the membrane, which leads to membrane spreading. For our further observations (Figure 3), the already spread *S. aureus* lipid membrane with *N*-alkylamide 3d incorporated into the bilayer forms the background of our images. Right after the spread of the *S. aureus* lipid membrane, we start observing attachment of *N*-alkylamide 3d molecules on top. The antibiotics attach in the form of sphere-shaped aggregates (Figure 3a,b, Figure S6b) that are sometimes mobile on the membrane surface and merge together into bigger sized spheres (Figure 3b). The size of all these aggregates is 2–13 nm in height (Figure S7).

The imaging is usually very noisy for the first 10–30 min after the membrane domains disappear and the membrane spreads. During that time, larger aggregates also attach on top of the membrane. One of the first such attachments is visible in white color in Figure 2c. This excessive amount of *N*-alkylamide 3d in time sediments on the membrane and transforms into supramolecular structures: carpets and rod-like aggregates (Figure 3c,d). We observed a step-by-step growth of the carpets over time (Figure 3c, Figure S8). There always appears to be a nucleation point where the carpet starts and then grows to all sides as new molecules of *N*-alkylamide 3d are attaching. Multiple layers of the carpets are often visible, with 5.2 ± 0.3 nm as an average height per layer. The rod-like structures could grow as single rods but also sometimes over each other or in a twisted form (Figure 3d). The single rod-like structures display a 4.8 ± 0.8 nm height. The height of structures that appear as two rods on top of each other (Figure 3d left and middle) is 9.0 ± 0.4 nm, corresponding to double the height of a single rod (Figure S9).

We interpret the supramolecular aggregates to be made of *N*-alkyamide 3d. We base this interpretation on the fact that *N*-alkyamide 3d itself can form elongated carpets and rod-like structures on mica alone. Moreover, we do not observe any membrane disruption or pore formation at any point of the *N*-alkylamide 3d activity on the *S. aureus* lipid membrane. This means that we do not observe any material removal from the membrane that could aggregate together with the excessive *N*-alkyamide 3d. However, there is a possibility that the aggregates are made of a mixture of lipids and *N*-alkyamide 3d. Studies using a scanning probe method, including chemical specificity, might allow for distinguishing between these possibilities.

## How Could *N*-Alkylamide 3d Activity Affect Bacterial Activity?

What are the possible consequences of the complex *N*-alkylamide 3d’s activity on living bacteria? First of all, the antibiotic invades the membrane and dissolves the membrane domains, which by itself is probably enough to kill the bacteria, as previously suggested.^6^ In addition, if we treat the *S. aureus* lipid membrane with 10–20 μg/mL *N*-alkylamide 3d, the membrane ends up being fully covered in a mix of carpets and rod-like structures. The covering of the membrane by the antibiotic in these structures could make the bacteria completely inaccessible to molecules from the outside and prevent signaling and waste disposal from the inside, even though it is not sure whether such a complete coverage also occurs *in vivo*. The concentrations in experiments when we see the domain dissolution are comparable to the MIC for *S. aureus*. The concentrations needed for the complete membrane coverage by the supramolecular structures is around 10 times the MIC and 10 times lower than the reported cytotoxic concentration.^21^ Such concentrations are common practice for clinical treatment of infections using membrane-active antibiotics like vancomycin or daptomycin.^35,36^

## Domain Dissolution Could Be a More Common Effect of Membrane-Active Antibiotics

Several modes of activity were proposed for membrane-active antibiotics that do not have a specific molecular target.^12,37^ The main two are formation of transmembrane pores and coverage of the membrane by carpets. Recently we reported on a new mechanism, the insertion of the antibiotic into the membrane and dissolution of membrane domains.^6^ Here, in the case of *N*-alkylamide 3d, we ultimately observed the carpet formation. In fact, our experiments with conventional AFM allowed us to capture the spreading of the membranes, followed by attachment of supramolecular structures such as carpets. The temporal resolution of the method, however, did not capture the influence on the membrane domains of the *S. aureus* lipid membrane. Only the use of high-speed AFM allowed us to observe the membrane domains and their dissolution.

We might, thus, hypothesize that also other antibiotics that are believed to form a carpet on the membrane and subsequently disrupt the membrane by pulling out micelles, such as aurein, citropin, or dermaseptin,^14,38−40^ could as their first step of activity insert into the membranes and affect the lateral organization. The use of complex membranes that contain the membrane domains and a method with a temporal resolution in a range of seconds is crucial to observe this new mechanism of antibiotic activity. Experiments with the antibiotic melittin also show that the membrane’s lateral organization is affected; however, after pores are formed, some domains are still visible.^6^ This shows that certain antibiotics are also likely to be able to exert their function without the complete dissolution of the membrane domains.

The membrane spread that is associated with the antibiotic insertion into the membrane (Figure 2e) can also be observed by standard AFM, if small supported membrane patches^14,41^ instead of fully covering supported membranes^22,42^ are used. However, molecule insertion into the membrane does not necessarily result in a large spread of the membrane as observed for AMC-109^6^ and here for *N*-alkylamide 3d. For instance, ethanol molecules also insert into the membrane and dissolve the membrane domains,^6^ but in the ethanol case this does not lead to spread of the membranes over the surface.^6,43^

To decipher the activity of the membrane-active antibiotic on a nanoscopic level, we might also use computer simulations and NMR experiments. In our study on the peptidomimetic AMC-109,^6^ molecular dynamic simulations predicted spontaneous formation of micelle-like aggregates in water before attacking the bacterial membrane and further visualized the process of the aggregate insertion into the membrane. The simulations of Bond et al.^44^ showed details of the orientation of the peptide maculatin inside a membrane and its concurrent coverage of the membrane. This peptide, hence, appears to be combining the pore-forming activity with coverage of the membrane surface, which was also later supported by NMR studies of maculatin localization in model membranes.^45^ Furthermore, recent studies on teixobactin showed how the combination of solid state NMR, molecular dynamics simulations, and high-speed AFM yield clear insights on its mode of action.^17^ Such combination studies,^6,17,20,46,47^ hence, bring invaluable insight into the molecular mechanism of membrane-active antibiotics.

Here, we report the unusual activity of *N*-alkylamide 3d, an antibiotic that combines the features of a lipid and a peptide. We show that this antibiotic interacts with the *S. aureus* lipid membrane similarly to the peptidomimetic AMC-109 and the antiseptics benzalkonium chloride and ethanol, where it dissolves membrane domains. After this first step, where the membrane became saturated with *N*-alkylamide 3d molecules, it gets covered with supramolecular structures formed by the antibiotic. We hypothesize that also other membrane-active antibiotics could potentially affect the membrane domains as their first step of activity. To observe this phenomenon, approaches with both high spatial and temporal resolution in combination with complex bacterial membrane compositions are needed.

## Data Availability

Data that support the finding of this work can be found in the manuscript and its Supplementary Information. Raw AFM images and full high-speed AFM videos in the raw format used in the Figures, together with cross-section data used for the statististics can be found in an online repository (doi: 10.5281/zenodo.13310151). Data from experimental repetitions are available from corresponding authors upon request.

## Supporting Information Available

Materials and Methods; Figure S1: directional growth of *N*-alkylamide 3d on mica as viewed by high-speed AFM; Figure S2: no specific interaction of *N*-alkylamide 3d with the surface of gold Au(111) as viewed by AFM; Figure S3: *S. aureus* lipid membrane on various substrates viewed by AFM; Figure S4: thickness of the *S. aureus* lipid membrane on mica; Figure S5: analysis of the area occupied by domains in the *S. aureus* lipid membranes before and after the exposure to 5 and 17 μg/mL *N*-alkylamide 3d; Figure S6: *S. aureus* lipid membranes on plasma-cleaned glass upon exposure to *N*-alkylamide 3d; Figure S7: histogram of the spherical structures’ height measured from the high-speed AFM data; Figure S8: carpet formed by *N*-alkylamide 3d on top of the *S. aureus* lipid membrane; Figure S9: histogram of the rod-like structures’ height (PDF)
Supporting video 1: Side-by-side comparison of the domains’ movement in *S. aureus* lipid membranes before and after the treatment with 5 μg/mL *N*-alkylamide 3d, with the time stamp denoting the frame rate (time resolution) of our imaging; the movie on the right starts 19:50 min after the addition of the *N*-alylamide 3d, and a snapshot of both movies is shown in Figure 2a and 2b (AVI)

## Supplementary Material

nl4c01873_si_003.pdf

nl4c01873_si_002.avi
