# Supplementary material for: Membrane-Active Antibiotics Affect Domains in Bacterial Membranes as the First Step of Their Activity
Source: Nano Lett. 2024 Aug 15;24(38):11800–7. doi: 10.1021/acs.nanolett.4c01873 (PMC11440642; doi:10.1021/acs.nanolett.4c01873)
Supplement: Supplementary file 1 — nl4c01873_si_003.pdf [file nl4c01873_si_003.pdf]

Supporting Information for

Membrane-active antibiotics affect domains in bacterial membranes as the first step of their activity

Adéla Melcrová[1], Christiaan Klein[1], Wouter H. Roos[1*]

[1] Molecular Biophysics, Zernike institute for Advanced Materials, Rijksuniversiteit Groningen, the Netherlands

*Correspondence: Wouter H. Roos w.h.roos@rug.nl and Adéla Melcrová a.melcrova@rug.nl

Methods

Materials

N-alkylamide 3d was a generous gift from Xin-Shan Ye and Youhong Niu from Peking University. The compound was obtained as a dry powder (purity >95% determined by analytical HPLC[1]). Molecular structure is shown in Figure 1a, M_w = 675 g/mol. The material was stored as a powder in -20°C. For usage in experiments, we dissolved N-alkylamide 3d in milliQ water, stored in +4°C in dark and used it for a maximum of 3 months.

Staphylococcus aureus cells, strain RN4220, NCTC8325-4 derivative, restriction deficient and cured of prophages[2], was a generous gift from prof. J. M van Dijl from University Medical Centrum Groningen. The cells were stored in -80°C.

Phosphate buffered saline (PBS buffer, pH 7.4) in the form of tablets for dissolution in milliQ water, HEPES powder, and poly-L-lysine powder were purchased at Sigma-Aldrich.

Gold coated mica with 200 nm layer of (111) gold was purchased from Phasis Sàrl. Glass coverslips (brand: VWS diameter 25 mm and Menzel-Gläser diameter 25 mm from VWR) were used. SiO_2–coated silicon wafers, which are silicon wafers with a silicon dioxide layer thermally grown on its surface were purchased from Biotain Crystal Co., China.

S. aureus lipid membrane extraction

Lipid extraction from *S. aureus* cells was done as reported previously[3]. In short, *S. aureus* cells were cultivated in a growth medium in 37°C until saturated growth. The cells were centrifuged at 2095 x g at 4°C, the growth medium was exchanged for PBS buffer, and centrifuged again. The wet cells were stored at -80°C until lipid isolation following the modified Bligh & Dyer extraction protocol[4]. The wet cells were washed in milliQ water, weighted, and dissolved in water/chloroform/methanol in ratios 0.8:1:2, where the water content was 0.8 ml per 1 g of the cells, and left stirring in 4°C overnight. The mixtures were then centrifuged at 900 x g at 4°C for 15 min. The supernatant was resuspended in a 1:1 chloroform/water mixture and left to phase separate at room temperature for 2 days. The bottom layer was collected and dried in a rotary evaporator. Finally, the dry lipid film was weighed and dissolved in chloroform to the stock concentration of 10 mg/ml and stored at -20°C.

Preparation of glass slides

Plasma cleaned glass: Glass coverslips were sonicated in acetone for 30 minutes, then in ethanol (97%) for 30 minutes, and in an aqueous KOH solution (1M) for 10 minutes. The cover slips were then washed in milliQ water and dried at 60°C overnight. The coverslips were plasma cleaned for 15 minutes using a plasma cleaner from Plasma Etch Inc., model PE-50, and used for the AFM imaging within 15 minutes after the plasma cleaning.

Poly-L-lysine coated glass: Coating of glass slides by poly-L-lysine was done as described in [5]. Glass coverslips were washed for 10 minutes in ethanol-HCl solution, washed twice with milliQ water, and placed for 1 h into 1 mg/100ml poly-L-lysine solution in milliQ water. The coated slides were then briefly washed with milliQ water and dried at 60°C overnight. The coated slides were stored at 4°C and used for a maximum of 1 month.

Conventional atomic force microscopy

Preparation of liposomes: *S. aureus* lipid extracts in chloroform were pipetted into the glass vial. The chloroform was evaporated using an argon stream and let dry completely for >1 hour in a vacuum. PBS buffer was added to the lipid film in the concentration of 0.3–1 mg/ml. To help the resuspension, the mixture was vigorously shaken for 1 minute followed by 5 cycles of freezing by liquid nitrogen and thawing in warm water. For the AFM experiments the resuspended liposomes were then extruded 21 times through 0.1 μm pores polycarbonate membranes (Avanti Polar Lipids). The liposomes were stored in 4°C and used for the maximum of one week.

The surfaces used for deposition of *S. aureus* lipid membranes for AFM imaging were muscovite mica, SiO_2–coated silicon wafers, gold Au(111), plasma-cleaned glass, and poly-L-lysine coated glass. For mica, SiO_2, gold, and poly-L-lysine coated glass the sample cell for AFM was prepared by gluing a small piece of surface of choice on microscope glass slide with a transparent epoxy glue (common household use epoxy glue). A glass ring was attached around it by two component biocompatible glue (Bruker Nano GmbH). The sample cell for plasma-cleaned glass surface was prepared analogically, except that we used a metal holder with a metal ring to create the AFM imaging well.

For all surfaces except of plasma-cleaned glass, the liposomes were diluted to 0.02–0.04 mg/ml in PBS buffer. 10 μl drop of the diluted liposomes was deposited on the surface and let to sediment for >10 min. In some cases, the PBS buffer was then washed out 5 times and replaced with a new 10 μl drop of PBS buffer to press the liposomes to the surface and support their collapse into supported bilayers. PBS buffer was then added to the total volume of 0.5–1 ml. In case of plasma-cleaned glass, we used 0.1–0.2 mg/ml liposomes to achieve full coverage of the surface with the supported membrane. 250 μl of the liposomes were deposited on the plasma-cleaned glass, where they were incubated for 30 minutes. Afterwards the solution was washed out 5 times and replaced with fresh PBS buffer. Total of 0.4 ml sample was used for imaging on glass using the metal holder and ring.

AFM imaging was performed with an JPK Nano Wizard Ultra Speed AFM. The experiments were performed at room temperature (22°C) using qp-BioAC cantilevers (NanoAndMore GmbH) with a nominal spring constant 0.06 ± 0.03 N/m and a silicon nitride tip with a typical tip radius of curvature smaller than 10 nm. The imaging force was ~80–100 pN in all cases. We first image untreated membranes and then add a concentrated solution of the antibiotic to achieve the desired final concentration in the sample. All experiments are performed in liquid.

High-speed atomic force microscopy

HS-AFM experiments were performed at room temperature (22°C) using an RIBM (Japan) machine in amplitude modulation tapping mode in liquid[6–9]. Short cantilevers USC-F1.2-k0.15 (NanoWorld, Switzerland) with a spring constant of 0.15 N/m, resonance frequency around 0.6 MHz, and a quality factor of ∼2 in buffer were used. The cantilever free amplitude was set to 1 nm, and the set-point amplitude for the cantilever oscillation was set around 0.8 nm. Images were taken at 1–5 s per frame. A mica surface of diameter 1.5 mm glued on top of a 5 mm high glass rod was used as the sample stage. The glass rod was attached to the scanner Z-piezo using nail polish. For the activity of N-alkylamide 3d on *S. aureus* lipid membranes, a 3 μl drop of 0.02–1 mg/ml liposomes in PBS buffer was deposited on the freshly cleaved mica. The liposomes were left to incubate for >15 minutes, the PBS buffer was replaced with the buffer containing Mg^{2+} ions (25 mM $MgCl_2$, 100 mM NaCl, 1 mM HEPES, pH 7.4) to rupture the liposomes and achieve high coverage of the mica surface with the membrane. After 5 minutes the buffer was washed out 5 times and replaced with PBS buffer, in which we performed the imaging. In some cases, the Mg^{2+} washing step was skipped. The scanner head was then put upside down into a small liquid chamber containing the cantilever and filled with 40–80 μl of the recording solution (PBS buffer). First, we image untreated membranes and then add 5 μl of the buffer with a concentrated solution of the antibiotic to achieve the desired final concentration in the sample.

The disappearance of the domains as reported in Figure 2 was observed in 6 independent high-speed AFM experiments. Spread of the membrane was observed in 12 independent experiments using both high-speed AFM and conventional AFM. Attachment of the supramolecular structures reported in Figure 3 on top of the spread membrane was observed in 6 independent high-speed AFM experiments on membranes supported on mica, and 3 conventional AFM experiments on membranes supported on plasma-cleaned glass.

The analysis of the membrane area occupied by the domains was done in ImageJ. The membrane area was cropped manually, and the threshold based on the median of the local brightness changes was applied. Domain area was measured at 2 separate times for all tested concentrations: 51.8% and 51.2% for 0 μg/ml, 48.0% and 48.0% for 5 μg/ml, and 39.0% at 15s and 24.3% at 22s after the increase to 17 μg/ml.

For the height measurements of the structures on top of the membrane, the mean height of the surrounding membrane was obtained from cross-sections, and subtracted from the height of the structure. The values in the results section are depicted as mean ± error of the mean of individual values. Statistics was done on N=28 (4 experimental days) for spherical aggregates, N=5 (4 experimental days) for carpets including the last layer of multiple-layered carpets, N=30 (4 experimental days) for single rods and N=6 (2 experimental days) for double-layered rods.

References used in the Methods section

1. Niu Y, Wang M, Cao Y, Nimmagadda A, Hu J, Wu Y, Cai J, Ye XS. Rational Design of Dimeric Lysine N - Alkylamides as Potent and Broad-Spectrum Antibacterial Agents. Journal of Medicinal Chemistry. 2018;61(7):2865–2874.

2. Kreiswirth BN, Löfdahl S, Betley MJ, O'Reilly M, Schlievert PM, Bergdoll MS, Novick RP. The toxic shock syndrome exotoxin structural gene is not detectably transmitted by a prophage. Nature. 1983;305(5936):709–712.

3. Melcrová A, Maity S, Melcr J, de Kok NAW, Gabler M, van der Eyden J, Stensen W, Svendsen JSM, Driessen AJM, Marrink SJ, Roos WH. Lateral membrane organization as target of an antimicrobial peptidomimetic compound. Nature Communications. 2023;14(1):4038.

4. Bligh EG, Dyer WJ. A Rapid Method of Total Lipid Extractions and Purification. Canadian Journal of Biochemistry and Physiology. 1959;37(8):911–917.

5. Vorselen D, Piontek MC, Roos WH, Wuite GJL. Mechanical Characterization of Liposomes and Extracellular Vesicles, a Protocol. Frontiers in Molecular Biosciences. 2020;7(July):1–14.

6. Ando T, Kodera N, Naito Y, Kinoshita T, Furuta K, Toyoshima YY. A High-speed Atomic Force Microscope for Studying Biological Macromolecules in Action. ChemPhysChem. 2003;4(11):1196–1202.

7. Uchihashi T, Kodera N, Ando T. Guide to video recording of structure dynamics and dynamic processes of proteins by high-speed atomic force microscopy. Nature Protocols. 2012;7(6):1193–1206.

8. Shukla R, Lavore F, Maity S, Derks MGN, Jones CR, Vermeulen BJA, Melcrová A, Morris MA, Becker LM, Wang X, Kumar R, Medeiros-Silva J, van Beekveld RAM, Bonvin AMJJ, Lorent JH, et al. Teixobactin kills bacteria by a two-pronged attack on the cell envelope. Nature. 2022;608(7922):390–396.

9. Maity S, Trinco G, Buzón P, Anshari ZR, Kodera N, Ngo KX, Ando T, Slotboom DJ, Roos WH. High-speed atomic force microscopy reveals a three-state elevator mechanism in the citrate transporter CitS. Proceedings of the National Academy of Sciences. 2022;119(6):e2113927119.

Supporting Figures

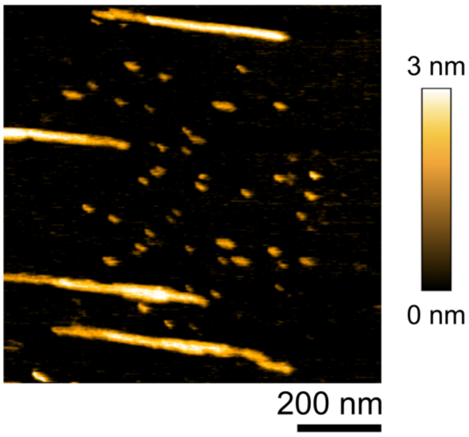

Figure S1: Directional growth of N-alkylamide 3d on mica as viewed by high-speed AFM. To perform this experiment, clean mica was used to which 10 µg/ml N-alkylamide 3d in PBS buffer was added. The image shows the growth of N-alkylamide 3d 15 min after the antibiotic addition. This behaviour was observed 5 times both at standard AFM (Figure 1) and high-speed AFM.

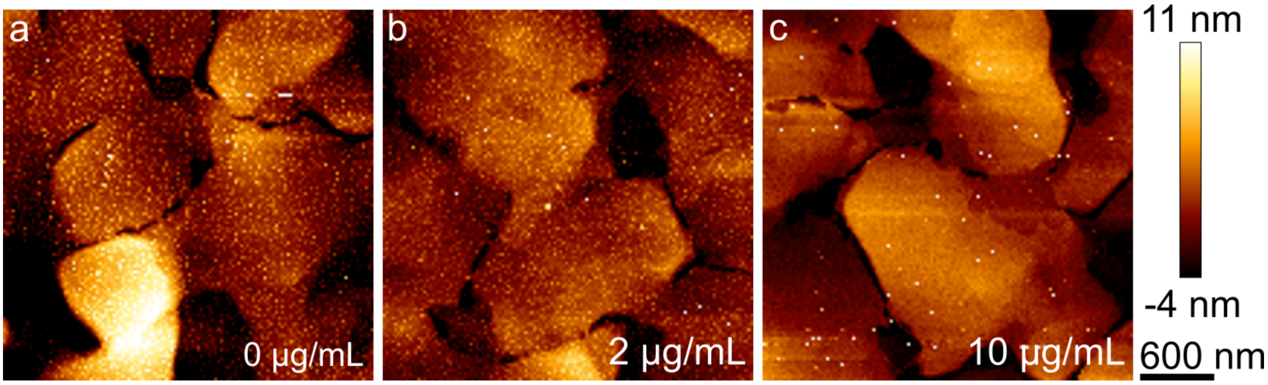

Figure S2: N-alkylamide 3d has no specific interaction with the surface of gold Au(111). **(a)** Gold surface imaged in the presence of pure PBS buffer and upon the addition of 2 µg/ml **(b)** and 10 µg/ml **(c)** N-alkylamide 3d. There is no aggregation of N-alkylamide 3d observed on the surface of gold. Captured with conventional AFM.

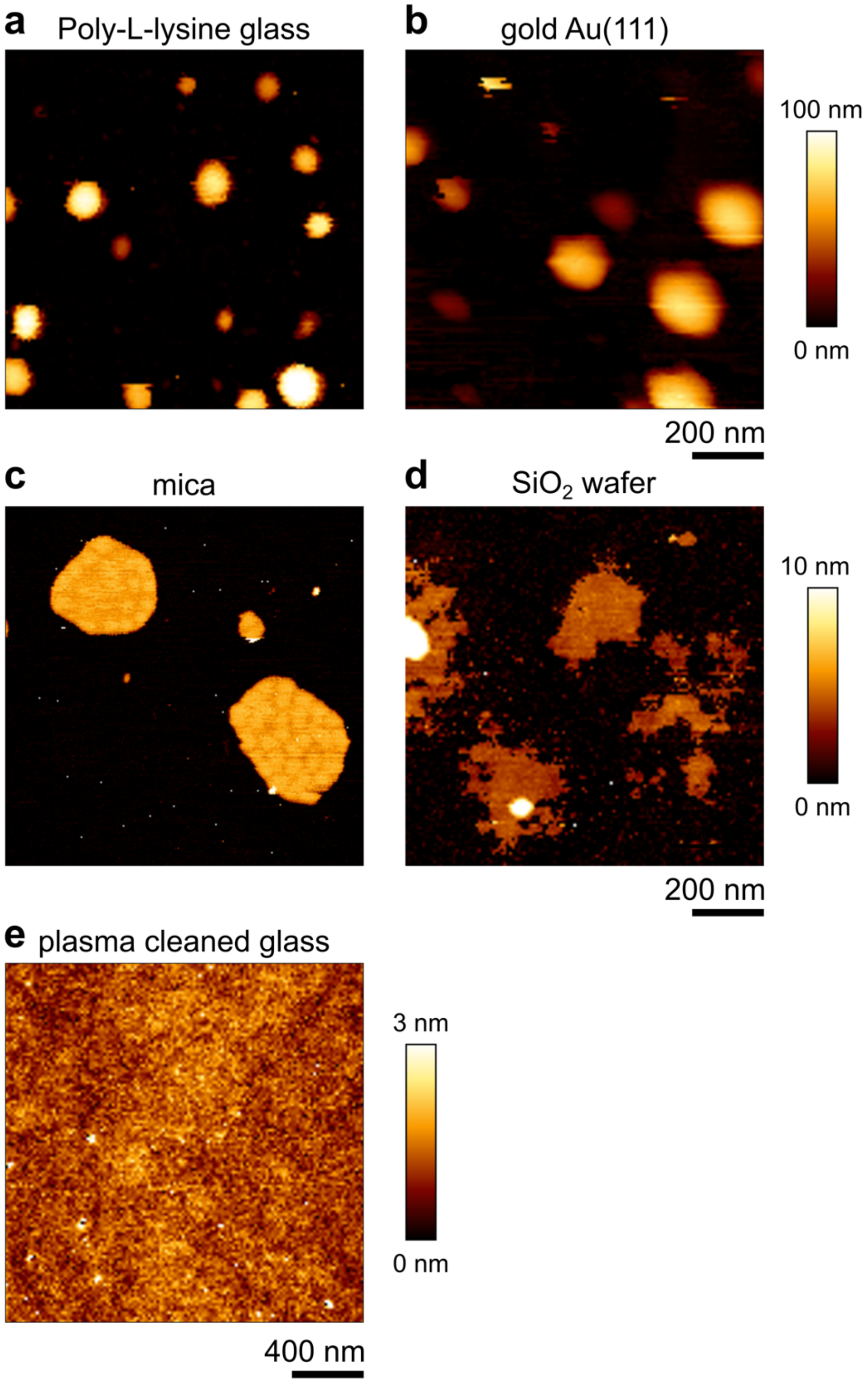

Figure S3: *S. aureus* lipid membrane on various substrates viewed by AFM. **(a)** Poly-L-lysine coated glass, **(b)** gold Au(111), **(c)** mica, **(d)** SiO_2 wafer, and **(e)** plasma-cleaned glass. Captured with conventional AFM.

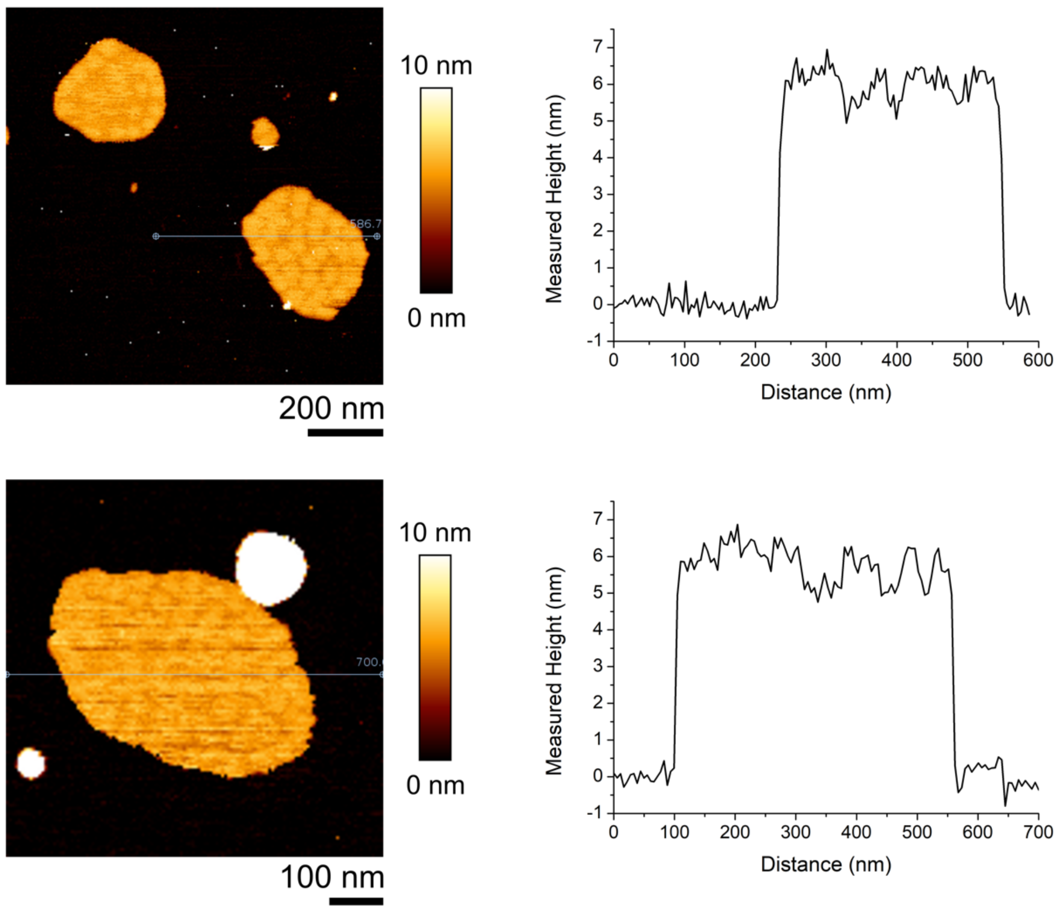

Figure S4: Thickness of the *S. aureus* lipid membrane on mica. Two examples of height images and corresponding cross-section line profiles showing the height of these membranes. We can recognise lateral domains with a bit higher thickness than the rest of the membrane. During the imaging with conventional AFM, with an imaging speed of 2.5 minutes per 128x128 pixels, the domains are blurry due to their lateral movement. The thickness on top of the domains is ~6 nm.

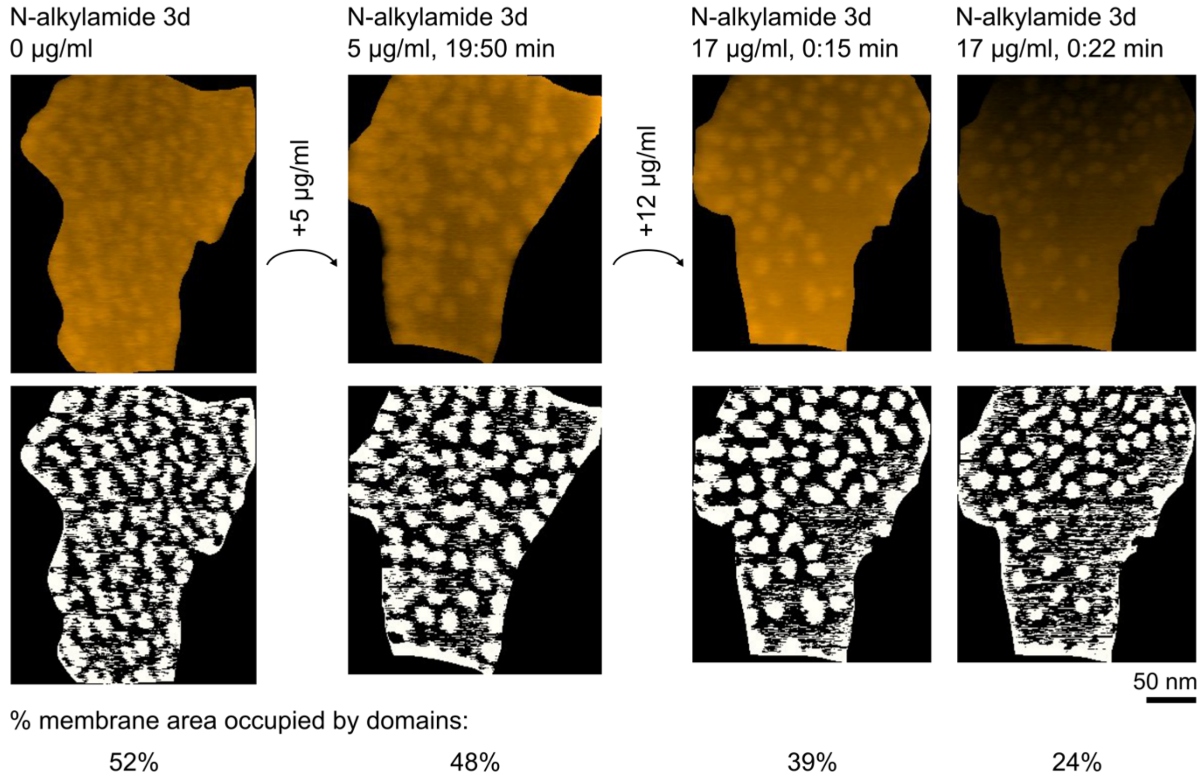

Figure S5: Analysis of the area occupied by domains in the *S. aureus* lipid membranes before and after the exposure to 5 and 17 µg/ml N-alkylamide 3d. High-speed AFM images where cropped to analyse only the membrane area. Threshold based on median of local contrast changes was used to identify the domains, and calculate their area. Area occupied by the domains was measured at 2 times for all N-alkyalmide 3d concentrations: 51.8% and 51.2% for 0 µg/ml, 48.0% and 48.0% for 5 µg/ml, and 39.0% at 15s and 24.3% at 22s after the increase to 17 µg/ml. The time stamps in [min:sec] denote the time after the addition of the stated antibiotic concentration.

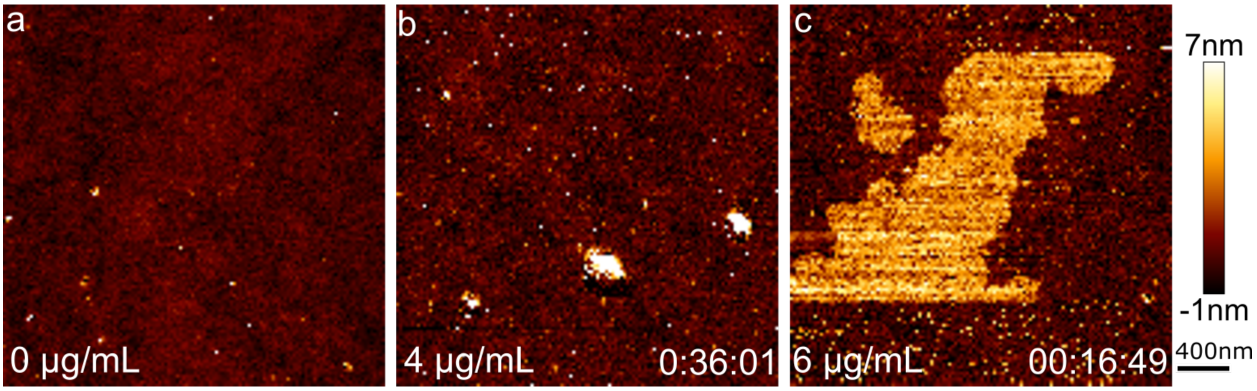

Figure S6: *S. aureus* lipid membranes on plasma cleaned glass as they go through 2 distinct stages after their exposure to N-alkylamide 3d. **(a)** Untreated *S. aureus* lipid membrane. **(b)** The membrane after the addition of 4 µg/mL N-alkylamide 3d with sphere-shaped aggregates of N-alkylamide 3d on top (white structures). **(c)** The membrane after the addition of 6 µg/mL N-alkylamide 3d with carpet formation. Time stamps [hour:min:sec] indicate the time after the addition of N-alkylamide 3d of respectively 4 µg/mL and 6 µg/mL. Figure is representative of two successful independent experiments using conventional AFM.

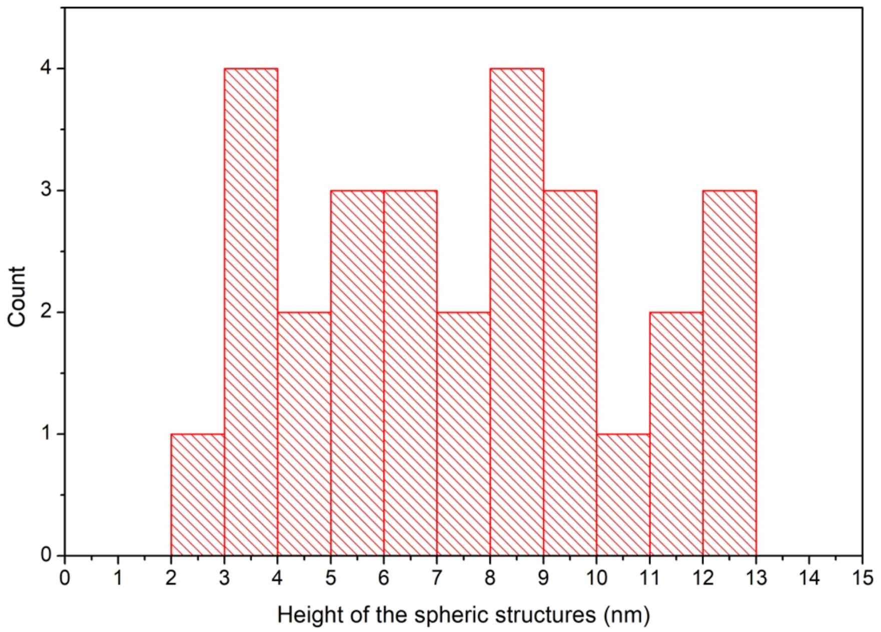

Figure S7: Histogram of the spherical structures' height. Measured from line cross-sections over the structures imaged by high-speed AFM. N=28, 7 ± 3 nm represents the mean and standard deviation of the mean values. Statistics over 4 experimental days.

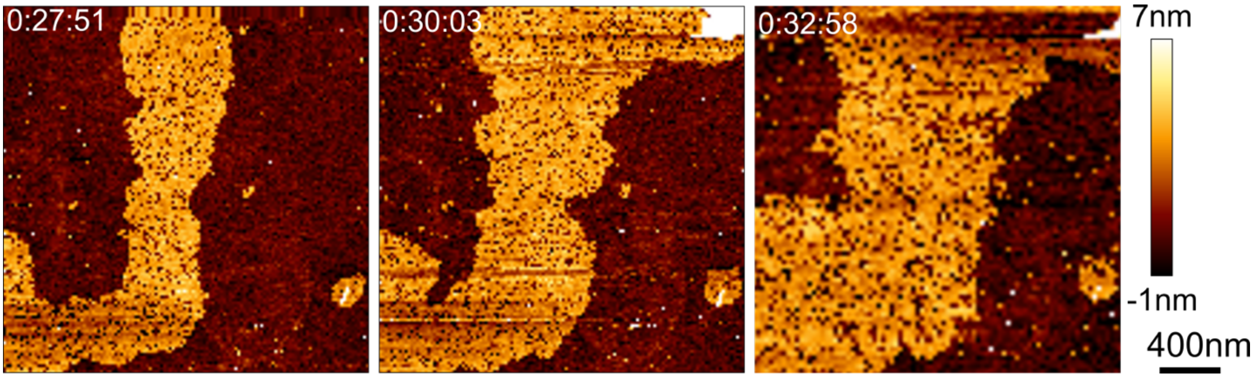

Figure S8: Carpet formed by N-alkylamide 3d (yellow) grows on top of the *S. aureus* lipid membrane (red). The membrane that is spread on plasma cleaned glass forms the background of these images. The N-alkylamide 3d carpet growth over the course of 5 minutes in the same location is followed. Captured with conventional AFM. Time stamps [hour:min:sec] indicate the time after the addition of N-alkylamide 3d at a concentration of 6 µg/mL.

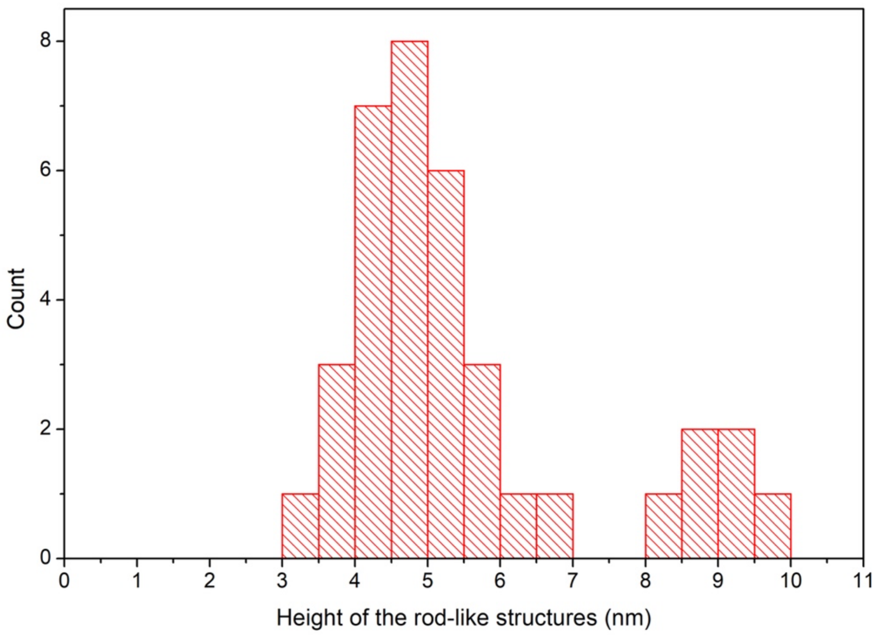

Figure S9: Histogram of the rod-like structures' height. Measured from line cross-sections over the structures imaged by high-speed AFM. Measured over 4 experimental days, N=36. The two populations in the histogram correspond to single rod-like structures and two of them stacked on top of each other. The average heights (mean ± standard deviation) of those two groups are 4.8 ± 0.8 nm and 9.0 ± 0.4 nm, respectively.
